# Responses to Offense at Work and the Impact of Hierarchical Status: The Fault of the Leader, Causal Attributions, and Social Support During the Covid-19 Pandemic Crisis

**DOI:** 10.3389/fpsyg.2021.734703

**Published:** 2021-11-26

**Authors:** Mihai Ion Marian, Karla Melinda Barth, Mihai Ionut Oprea

**Affiliations:** ^1^Psychology Department, University of Oradea, Oradea, Romania; ^2^Educational Sciences Department, University of Oradea, Oradea, Romania; ^3^Department of Sociology and Social Work, University of Oradea, Oradea, Romania

**Keywords:** revenge, reconciliation, attributional style, social support, emotions

## Abstract

The study explores the mechanism by which unadapted causal attributions and the perception of social support stimulate revenge and reconciliation at the social and professional level in the context of the current pandemic. In particular, the purpose of the study is to investigate the relationship between the accused, the victim and offender status and the search for revenge or reconciliation following a personal offense. To test the suggested research model, we analyzed the data collected by 167 (*m* = 28.52; SD = 8.98) employees in different organizations using a multifactorial experimental design. The results support the influence of attributional predictions in forming revenge and reconciliation and show that they are involved in the decision to carry out revenge, but especially in the way the employee interprets the trigger situation. In conclusion, the revenge is based on a negative attributional mechanism that produces the greatest deficit of adaptation to the situation and a weakening of the perception of social support, while reconciliation seems to be based on a much more complex socio-occupational mechanism. Leaders should pay attention to organizational communication during a crisis as they could encourage hopelessness depression. Adjusting crisis communication is crucial to ensuring job satisfaction that could mitigate negative effects.

## Introduction

We are living in times of great complexity to which there is currently no clear answer. The various and often contradictory explanatory trends on the pandemic declared by the WHO (World Health Organization) in 2020–2021, in association with often irrational government decisions, but justified by lack of knowledge of reality and supported by organizations and institutions (including sponsored media), have generated various (often negative) reactions in the case of employers, but especially employees. Organizational theory in this pandemic context is subject to serious and relevant verification at least at the application level. Is it possible that theoretical restructuring and a refinement of the implementation of the now-classic explanatory models is needed? Time will tell us whether the lesson has been learned or not.

In this context, Huynh (2020b) examining the role of socio-economic factors and the use of social networks in the perception of the risk generated by COVID-19 stressed that depending on geographical regions and social networking behaviors they can have a positive impact on the perception of the pandemic risk generated by COVID-19. In addition, Ramkissoon (2020, 2021b) and Huynh (2020b) indicated that understanding of the risk at Community level and adherence to the pro-health adaptive behavior promoted by public health institutions on COVID-19 may reduce the spread of the virus.

Cultural factors play an important role in controlling the spread of COVID-19 and limiting the pandemic. Huynh (2020a) considers fundamental cultural values and their incorporation into the transmission of information (which would reduce uncertainty) on limiting public gatherings in a pandemic context to be important.

Our focus is on revenge and reconciliation, but without ignoring the need to change the social and professional habits that are important in promoting healthy lifestyles. As Ramkissoon (2020) “COVID-19 is leading us to re-consider our existing behaviors with opportunities to embark on new designs for a sustainable future” (p. 7).

At the beginning of the century, vengeance in the workplace was given attention in studies specific to organizational psychology (Skarlicki and Folger, 1997; Bordia et al., 2014), so research focused on how the incentives of vengeance can motivate extreme workplace behavior, such as employee theft (Greenberg, 1990), anti-social behavior (Sanders and Hamilton, 2001; Marian and Filimon, 2010), rumors (Bordia et al., 2014), aggressiveness and violence (Sanders and Hamilton, 2001). However, employees who play a central role in the performance of firms are largely ignored in the literature and in an analysis of published studies we see that around 5% focused exclusively on employees (Larson et al., 2008; Aguinis and Glavas, 2012).

### Socio-Cognitive Aspects of Vengeance and Reconciliation

The current study is an attempt to address this gap by examining how employees’ perceptions of the top level offensive can generate vengeance or reconciliation in firms, which will shape future social attitudes and behaviors.

We consider revenge to be an action in response to perceived damage that is intended to bring punishment to the person considered to be responsible (Marian and Filimon, 2010). Forgiveness, on the other hand, is an action in response to the perceived damage or offense of another person. In other words, a deliberate decision by which the victim gives up the anger, resentment and desire for punishment of the person who was responsible for the harm caused (Greenberg, 1990; Sanders and Hamilton, 2001).

We have seen so far that literature is poor in terms of empirical studies on the influence of a firm involvement in mitigating small conflicts that can change employee attitudes and behavior in the workplace (Peterson et al., 1993; Farooq et al., 2019; Rungduin et al., 2019).

Does revenge always have negative effects? Empirical evidence shows that vengeance prevents abusive behavior by directors (Aquino et al., 2001) and acts as a catalyst for positive change in organizations (Rungduin et al., 2019). Although vengeance can sometimes lead to positive effects in organizations, it often leads to counteroffensive (Sanders and Hamilton, 2001; Bordia et al., 2014), resulting in an escalation of the already established conflict.

Revenge is a part of the social fabric of the organization’s life and is also at the heart of organizational conflicts, which may be motivated by concern for lack of justice, such as organizational policies (Bradfield and Aquino, 1999; Aquino et al., 2001).

Focused research on organizational justice identified vengeance as a return of the feeling of justice (McCullough et al., 1997, 1998) but without sufficient empirical support of the social cognitive dynamics of vengeance at work (Aquino et al., 2001; Fletcher and Weinstein, 2002). In our study we want to fill the gap in the literature, in addition we want to check how employees will react when the offending has a higher rank. The social cognitive paradigm of helplessness learned in this context will provide us with the essential elements that will be the core around which we will build the explanatory model (Peterson et al., 1993).

Forgiveness and reconciliation seem not to have received sufficient attention in empirical research on management (Enright et al., 1989; Kim et al., 1998; Fletcher and Weinstein, 2002) however, qualitative research into workplace revenge suggested that forgiveness (Sanders and Hamilton, 2001; Post, 2008) is a possible response of employees, which restores the feeling of justice. Forgiveness-oriented behavior occurs with a higher probability compared to revenge-oriented behavior (Aquino et al., 2001; McCullough et al., 2013) when employees focus their cognitive energy (Peterson et al., 1993; Marian and Filimon, 2010) on forgiveness. Literature does not clearly record the role played by perceived social support, so is it likely to have a strong effect?

Forgiveness can be expressed interpersonally by reconciliation, as an attempt by the victim to restore a deteriorated relationship by extending goodwill actions to the offender (Rusbult et al., 1991; McCullough et al., 1997). In our study, we focus on reconciliation because it is a behavioral expression of forgiveness and may have a direct effect on social relationships in which the causal attributional mechanisms of employees could be identified (Peterson et al., 1982, 1993; Zimet et al., 1988).

As we have seen so far, organizations are arenas where vengeance and forgiveness often occur, we have little information about the factors that influence the choice of vengeance or the effort to reconcile with the offending at work. Among the most important organizational variables that could influence revenge or reconciliation are those associated with power and status (Peterson et al., 1993; Kim et al., 1998; Aquino et al., 2001; McCullough et al., 2013). The asymmetry of power between the victim and the offender was postulated to influence the staged revenge (Kim et al., 1998).

Persons with high status comply with social rules and consider revenge to be immoral or non-professional (Aquino et al., 2001; McCullough et al., 2013). Although vindictive revenge could be considered a counteroffensive for people with higher hierarchical status, controlling revenge could be regulatory (Kim et al., 1998; Aquino et al., 2001; Post, 2008; McCullough et al., 2013).

On the basis of our comments, we believe that people with high status are much more reconcilable and benevolent with their offensives than those with an additional lower status, highly-ranking employees consider that they have more to gain by demonstrating their compassion (Zimet et al., 1988).

In the review of revenge and reconciliation, we have set out: (a) to define vengeance and forgiveness in functional terms that make them more susceptible to adaptive analysis; (b) to describe the mechanisms that give rise to revenge and forgiveness; and (c) highlight the potential causes and providing the methodological approach that will be presented later in this study.

We have set out to investigate the relationship between the accusation, the status of victim, and offender, and the search for revenge or reconciliation following a personal offense. We examine the effects in relation to the hierarchical differences of the offending. The study aims to provide much more concrete data on the effects of the power and status variables, secondly, we measure key variables such as causal attributions (Abramson et al., 1989; Peterson et al., 1993) that could motivate revenge or reconciliation at work.

Hypothesis 1: Negative attributional style, negative emotions, and perceived support are preachers of revenge-oriented behavior.
Hypothesis 2: Perceived social support, hierarchical status, and attributional style are preachers of reconciliation-oriented behavior.

We assume that the asymmetry between the offender and the victim may influence revenge, on the other hand we assume that the victim’s and offender’s status, and the victim’s hierarchical status act as possible moderators of the relationship between causal attributions and the adopted reconciliation or revenge behavior.

Hypothesis 3: The relationship between causal attributions and vindictive behavior is low when the offender has a higher status than the victim compared to the situation when the offender has an equal or lower status.

We have seen that the literature focuses primarily on testing the connection between the cognizance and the perceptions of the victim and the type of vengeance or forgiveness response. By testing the relationship between the causative inappropriate attributions and the forgiveness and vengeance responses we explore possible moderators of this relationship which is another objective of our study.

Hypothesis 4a: The relationship between the offending situation reported by employees and revenge-oriented behavior is mediated by attributional style.

Hypothesis 4b: The relationship between offensive and revenge is mediated by perceived social support and negative emotions.

## Materials and Methods

### Participants

The questionnaires were applied to 167 employees. The average age of respondents was 28.52 (SD = 8.98), of which 46 (27.5%) were men and 121 (72.5%) women. In terms of qualification, the majority of respondents received a baccalaureate degree (59.9%), a bachelor degree (31.1%), and a low number of graduates in vocational schools (9%). The majority of respondents did not lead the organization (71.5%). The average age in the organization of these employees was 5,56, which varied between 1–21 years.

### Measures

*Attributional Style Questionnaire* (ASQ) devised by Peterson et al. (1982); it is a measure of explanatory style patterns which in turn reflects ones tendency to select certain causal explanations for favorable or unfavorable events. The internal consistency reported by the Marian (2010) was 0.82 for positive events, and 0.72 for negative events. This moderate internal consistency is supported by other findings (Marian and Filimon, 2010).

*Multidimensional Scale of Perceived Social Support* (MSPSS) devised by Zimet et al. (1988); it consists of 12 items loaded on three factors: (a) family, (b) friends, and (c) significant others. Each item is structured according to the three factors. Internal consistency is 0.91 (12 items). Test-retest trust quotient of the two testing phases (T1 and T2) is between 0.67 and 0.80 (Marian, 2006).

*Profile of Mood States* (POMS) was accepted as an efficient way of measuring psychological stress. This study evaluated the psychometric properties of a shorter, 20-item version of the POMS. For all samples, internal consistency estimates for the POMS subscales were comparable to those for the original POMS (internal consistency is 0.90 for negative emotions and 0.88 for positive emotions; test-retest trust quotient is between 0.32 and 0.56) (Marian, 2007). The POMS is considered an alternative to the original POMS when a brief measure of psychological distress is desired.

#### Revenge Scale

The scale was to be developed by Wade in 1989 (Bradfield and Aquino, 1999; Aquino et al., 2001) and translated into Romanian (Marian and Filimon, 2010) demonstrating validity and reliability in determining the levels of intent to revenge the participants. The scale consists of five statements on a Likert scale from 1 to 5. The internal consistency of the range scale on the Romanian population is 0.82.

#### Reconciliation Scale

From the scale of Wade in 1989 (Bradfield and Aquino, 1999; Aquino et al., 2001). I took four Items that measured the building reconciliation on a Likert scale from 1 to 5. The items measure the extent to which a victim makes efforts to repair or improve the relationship with the offending as a result of the offensive. The scale was translated into Romanian (Marian and Filimon, 2010) and previously validated indicating good internal consistency (0.72).

The *hierarchical status* of the victim and the offender. Participants indicated the rank of the offended using one of the following words: “Subordinate”, “direct boss”, “administrator”, and “colleague”. In subsequent statistical processing we combined the top hierarchical positions in a single category of offender (code “0”) with a status higher than that of the victim and the subordinates and colleagues or those with equivalent status entered the second category (code “1”).

#### Controlled Variables

A number of variables which we believe that, at least theoretically, they could be linked to dependent variables, in this way, we control age because there is evidence in the literature that older people are more sophisticated in the moral judgment of forgiveness and as a result, they are much more willing to see reconciliation as an appropriate response to perceived inequity. We also control the similarity of gender and ethnicity as a result we use dummy variables.

### Procedure and Data Analysis

The data used in this study was part of a broader study on labor relations. The questionnaires were administered to employees in different institutions. The questionnaires used in the research were applied by the investigators without knowing the purpose of the research in order not to influence (hypothetical) the respondents. Participants were volunteers and retained anonymity by using a code or pseudonym code on the answer sheets. Researchers have tried to obtain the most accurate information possible on current workplace experiences, such as unpleasant or offensive experiences. In the first phase of the research, research participants have been interpolated about possible offenses from other employees in the workplace in the last month. Employees who recalled an incident and agreed to participate in phase two research were instructed to complete the questionnaires. The instructions provided that the participants would fill in the questionnaires according to the incident presented and note whether the offended person was in a higher, equal or lower position. In phase three respondents assessed the intensity of the offensive on a scale from 1 (very little) to 10 (very much). The questionnaires were related to behavioral and cognitive responses as well as the consequences of the offensive.

### Data Analysis

The results obtained were analyzed in the statistical pack for social sciences (SPSS). In the first phase, incomplete information was removed and calculated using descriptive statistics and research assumptions were tested in phase 2, ANOVA test, *t*-test, correction analysis, regression, and the structural equation in the case of causal model for recovery tested by AMOS program.

The AMOS version 17 program was used in the construction of the explanatory model of the revenge (Structural Equation Modeling; SEM). Unlike other approaches to assess a mediation model (Baron and Kenny, 1986), SEM is more appropriate because Type-I-errors and statistical power (Mackinnon et al., 2002; Kline, 2011) could be better balanced by a simultaneous test of Sinification of both the independent variable to the mediating variable, as well as the path from the mediating variable to the dependent variable.

## Results

Firstly, we conducted a confirmatory factor analysis (CFA) on structure of Revenge and Reconciliation Scales using AMOS. We hypothesized a two-factor model to be confirmed in the measurement portion of the model. We evaluated the assumptions of multivariate normality and linearity through SPSS.

The final sample size was 167 and there were no missing data. Goodness of fit indices included the chi-square, the comparative fit index (CFI), Tucker-Lewis Index (TLI), and the root-mean-square error of approximation (RMSEA).

**FIGURE 1:**
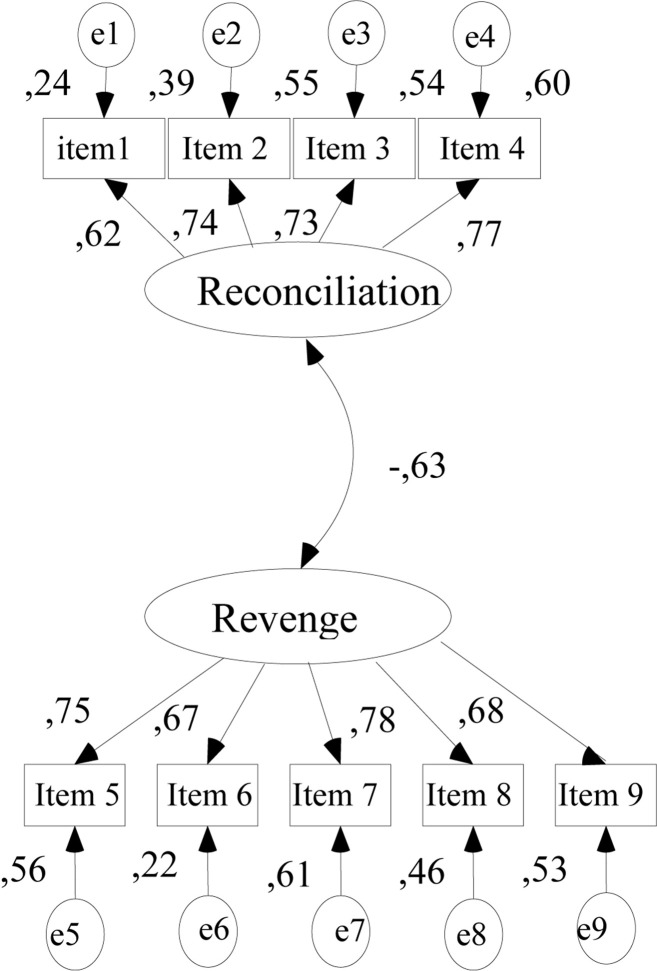
Confirmatory factor analysis revenge and reconciliation scale.

Our initial results based on CFA indicated an adequate model fit of the tested models corresponding to the factor structure of Revenge and Reconciliation Scales. The comparative fit index (CFI) = 0.90, the Tucker-Lewis fit index (TLI) = 0.88, and the RMSEA = 0.07, χ^2^ = 545.247, *p* < 0.01. Similar with Aquino et al. (2001), the items are loaded on factors revenge and reconciliation (Figure 1).

Recorded demographic variables have not generated differences (test *t*; *p* > 0.05) for sex, civil status, level of education in revenge and reconciliation. Therefore, in the statistical processing of data, we will not return to these issues. Descriptive statistics and correlations between study variables are presented in Table 1. Social desirability has been controlled in all statistical analyses.

**TABLE 1:** Descriptive statistics and correlation test between the scale scores.

**Variable**	***M***	**SD**	**1**	**2**	**3**	**4**	**5**	**6**	**7**
1. Revenge	9.55	3.80	–						
2. Reconciliation	14.73	2.90	−0.32**	–					
3. Intern causal attribution	4.49	0.58	0.20**	–0.03	–				
4. Stable causal attributions	4.17	0.63	0.23**	0.02	0.28**	–			
5. Global causal attributions	3.69	0.89	0.03	0.09	0.04	0.52**	–		
6. Social support	70.25	10.57	−0.19*	0.61**	0.02	0.14	0.28**	–	
7. Positive emotion	35.74	4.44	0.02	0.21**	0.15*	0.27**	0.31**	0.42**	–
8. Negative emotion	20.82	5.39	0.26**	0.00	–0.08	0.10	0.36**	–0.00	0.11

*p < 0.05; **p < 0.01.

We estimate two regression models in examining the mediating role of motivation for revenge and reconciliation in line with the proposed objectives.

### Relationship Between Causal Attributions, Emotions and Intent to Revenge Employees

According to the first hypothesis, the results show a positive relationship between the negative causality attributions (β = 0.19; *p* < 0.01; rp = 0.19) and the intention to revenge (Table 2), which points to a shift toward vindictive behavior in the context of the reduction of employee social support in non-usual situations (e.g., the current pandemic with global events) beyond the effects of social desirability.

**TABLE 2:** Results of regression equation for predictive purpose in the case of revenge behavior.

**Variables**	**β**	***t***	***p***	**r_*p*_**
Positive emotion	0.36	0.434	0.66	0.03
Negative emotion	0.23	3.140	0.002**	0.23
Perceived social support	–0.26	–3.214	0.002**	–0.23
Negative attributional style	0.19	2.506	0.01*	0.19
*R* ^2^	0.15			

F_(4,162)_ = 5,467, *p* < 0.001; Dependent variable: revenge; *p < 0.01 and **p < 0.001; rp = Correlations part.

Negative emotions (β = 0.23; *p* < 0.002; rp = 0.23) and not positive emotions (β = 0.36; *p* > 0.10) are predictive for retaliation (Table 2), which means that emotions such as anger or anger become relevant to manifestation in a pandemic context (or a state of crisis for which there is no equivalence).

In an attempt to capture the effect of the perception of social support in relation to revenge (Zimet et al., 1988; Aquino et al., 2001; Marian and Filimon, 2010) it indicates a surprising aspect that we identify a decrease in the statistical indicator of the perception of social support that is predictive for vengeance (β = −26; *p* < 0.002); rp = −0.23) in the pandemic context generated by COVID-19.

We believe that negative causal attributions, negative emotions and the perception of social support formulated hypothesis (*R*^2^ = 0.15; *F*_(__4_,_162__)_ = 5,467, *p* < 0.001) which means that revenge does not tend to chronic but rather tends to become widespread in conflicts that can be framed in the context of work done, *R*^2^ indicates 15% coverage of the variant.

### The Relationship Between the Perception of Social Support, Causal Attributions of the Hierarchical State and the Reconciliation Behavior

We were proposing to look at the predictive role of causal attributions, social support and the hierarchical status of the offender in generating reconciliation behavior. Thus, in Table 3 we show that 46% of the dispersion of results in reconciled behavior (*R*^2^ = 0.46; *F*_(__4_,_162__)_ = 24,792, *p* < 0.001) can be explained by preachers identified in the study and partly supported by previous research (Stuckless and Goranson, 1992; Aquino et al., 2001; Marian and Filimon, 2010).

**TABLE 3:** Results of regression equation for predictive purpose in the case of reconciling behavior.

**Independent variables**	**β**	***t***	***p***	**r_p_**
Negative emotion	–0.12	–2.011	0.04*	–0.11
Hierarchical status	–0.08	–1.379	0.07	–0.07
Perceived social support	0.51	8.367	0.001**	0.48
Negative attributional style	–0.14	–2.364	0.02*	–0.14
*R* ^2^	0.46			

*F*_(4,162)_ = 24,792, *p* < 0.001; Dependent variable: reconciliation; *p < 0.05; **p < 0.01; rp = Correlations part.

In Table 3 the perception of social support (β = 0.51, *p* < 0.001; rp = 0.48) is positively associated with the behavior of reconciliatory, therefore we are deducing that employees who perceive social support at a high level and who have been offended even if the negative emotions were inherent (β = −0.12, *p* < 0.04); rp = −0.11) will seek to resolve the conflict without escalation (12, 33). Surprisingly, employees who are victims of an offense and who are at lower levels in the workplace have been more likely to resort to reconciliation (β = −0.08, *p* < 0.07; rp = −0.07) than those at levels higher or equal to the offending. In association with previous results, causal attributions as expression of personality traits (e.g., skills) have a negative relationship with reconciliation (β = −0.14, *p* < 0.02; rp = −0.14) which supports the fundamental role of dysfunctional cognizance in the training and cementing of socio-professional deficiencies in an organization (Peterson et al., 1993; McCullough et al., 2013).

### The Relationship Between the Negative Attributional Style and the Hierarchical State of the Offender Over Vindictive Behavior

We used a type 2 × 2 experimental bifactorial design, with the offender’s hierarchy status and the negative causal attributions (low vs. high) as independent variables (IV) and revenge oriented behavior as a dependent variable (DV).

**FIGURE 2:**
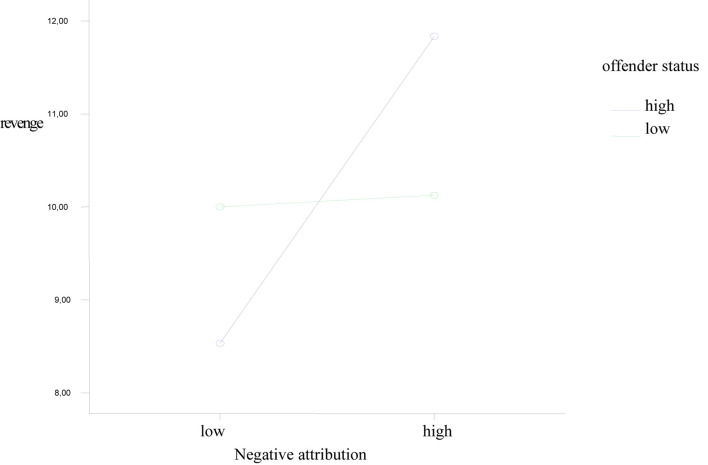
The interaction between that offender’s status and the negative causal attributions.

We claim from the assumption that the relationship between negative causality attributions and vindictive behavior is low when the offender has a higher status than the victim compared to the situation where the offender has an equal or lower rank. The employees’ scores were dichotomised at the CN (negative composite) scale of the ASQ (Peterson et al., 1993; Marian, 2010). The results of the comparison show that the offensive’s hierarchical status [*F*_(1,165)_ = 0.992, *p* > 0.11] does not generate direct revenge while attributional negative style [*F*_(1,165)_ = 7.259, *p* < 0.01] it has a dominant role as well as the interaction between the status of the offending and the negative attributional style [*F*_(1,165)_ = 5,729, *p* < 0.05]. The mediation effect shows that the victims of an offense with a lower position in the organization were willing to behave in a way that would reconcile with employees with a leading position (Figure 2). However, taking into account the current pandemic context as well as restrictions on the exercise of the profession (health limitations imposed by government and organizations) and the lack of obvious research in this context, we are looking at the results obtained with reservations.

### Testing Model

The hypothesis was tested with the structural equation modeling (SEM) using AMOS version 17. We test the relationship between the offending situation reported by employees and the retaliation-oriented behavior, but also the mediating role of the negative attributional style. On the other hand, we believe that the relationship between the offensive and revenge situation is mediated by perceived social support and negative emotions.

**FIGURE 3:**
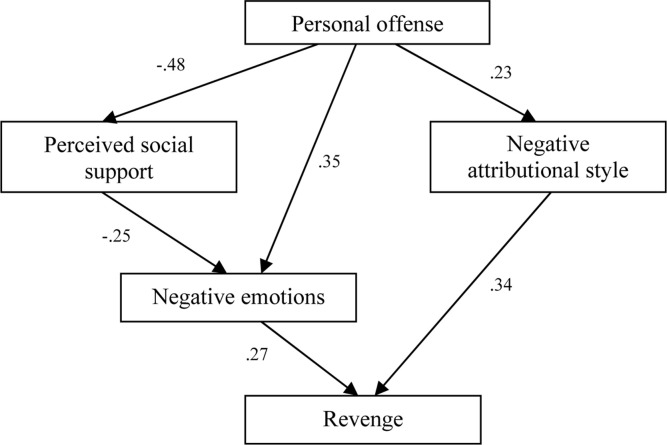
Structural model for revenge.

The hypothetical research model produced a good match with the data (χ2/df = 5.595; CFI = 0.97; TLI = 0.95; RMSEA = 0.07). The direct effects obtained in this model are shown in Figure 3. The four dimensions (exogenous variables) appear as strong vengeance preachers at the organizational level.

The negative attributional style had a strong effect on revenge (β = 0.34; *p* < 0.01) playing a mediating role between personal offense and the tendency toward revenge. The offensive situation has an important effect on the attributional style (β = 0.23; *p* < 0.02) and the causal interpretation will shape the subsequent response. In Figure 3 we show that the offensive situation will shape the emotions (β = 0.35; *p* < 0.01) that will average the relationship between the offense brought and the behavior oriented toward revenge. Based on the proposed model (Figure 3) we notice that an employee in an offensive workplace situation is likely to make offensive decisions based on the previously formed attributional style and emotional reactivity (β = 0.27; *p* < 0.02) that can be mediated by perceived social support (β = −0.25; *p* < 0.001).

We have double mediation in the relationship between interpretation of the offensive and revenge situation in the workplace that supports the results presented above (Figure 3). Thus, a strong effect is the perceived social support on the perception of the offense (β = −0.44; *p* < 0.01), but also on the negative emotions which partly justifies the assumption that hierarchical status could play an important role, but also the role of buffer of the perception of others in engaging behavior in response to the offense.

In the organizational context, the perception of the causes of the negative (i.e., offensive) situations as stable by their effects is not associated with the social self-image.

As we show in this research, reconciliation probably has much more sophisticated mechanisms for why the variables used in this study did not surprise by using experimentally satisfactory SEM relationships.

## Discussion

Unlike previous studies, current research is based on the social exchange perspective of employees in response to the perception of communication and the organization’s responsibility. Moreover, the study also established that organizational confidence is a result of the causal explanatory mechanism that can generate learned helplessness (Abramson et al., 1989; Peterson et al., 1993) in the absence of the buffer role of social support (Zimet et al., 1988; Marian, 2007; Miklósi, 2020).

Once again, we found support for the idea that the hierarchical status of the offending can stimulate the intention to revenge on the organization for unfavorable treatment (in the form of a breach of the psychological contract).

The motivation of revenge played a stronger role for employees who had a normative conviction that the unfavorable treatment (here offense) should be controlled by employees with a higher rank. The results were reproduced by recalling a self-assessed offensive situation.

Revenge can have several purposes (Kim et al., 1998; McCullough et al., 2013). Firstly, it may be an attempt to restore balance, secondly, revenge may be an attempt to teach the offending a lesson on the violation of the established rules and possibly an assurance that it will not go unpunished. Thirdly, revenge can be adopted to restore self-esteem or personal valorisation.

The relationship between causal attributions (as an expression of helplessness learned), revenge and reconciliation but especially the relationship between blemish and revenge provides additional information supporting studies indicating the integration of causal attributions in the analysis of revenge and forgiveness or reconciliation (Weiner, 1985; Abramson et al., 1989; Peterson et al., 1993).

We support the influence of causal attributional dictators on revenge and reconciliation that impact on the decision to carry out revenge, but especially on the employee’s interpretation of the offense. We believe that future attempts to take revenge will not improve the existential situation of employees but lead to a distorted perspective of social and intra-organization relationships (Zimet et al., 1988; Marian, 2006; Barattucci et al., 2017) associated with alienation, loneliness toward fellow humans where the result could be hopelessness depression as claimed by Abramson and his collaborators (Abramson et al., 1989; Peterson et al., 1993).

In Figure 3, it is possible for individual and organizational factors to determine acts of violence (revenge) (Sanders and Hamilton, 2001; Farooq et al., 2019; Teodora, 2019). Individual factors include differences in personality, so employees with high negative affectivity and hypothetical low affectivity tend to retaliate against perceived inaction. During the course of the study we focus only on the factors that amplify the connection of blame – revenge.

People who are in an offensive situation first try to explain what will crucially influence how they will continue to act. When the features attributions are frequent and the offense is assigned to another source without offense being seen in terms of permanence, personal and generalized, people will feel rather energetic and willing to settle the conflict, the dysphoric provision therefore mitigates what will not happen in the event of revenge where the negative emotional halon will persist.

Our study provides much more concrete data on the effects of the variable status (Figure 2), secondly, we measure directly variables (e.g., negative causal attributions) that could motivate vengeance in the workplace at least in unprecedented crisis situations (e.g., current pandemic situation). Testing the effects of the offending status differences in a natural environment we consider to be superior to the laboratory experiment.

Individuals offended by high status individuals are less likely to be targeted at seeking revenge (Bradfield and Aquino, 1999; Aquino et al., 2001). In addition, we believe that the status of the victim and the status of the offender are two independent constructions. They are theoretically distinct because the status reflects the strength of the relationship between the victim and the offender in a specific situation (Skarlicki and Folger, 1997; Bradfield and Aquino, 1999; Aquino et al., 2001).

On the basis of the theoretical arguments (Bradfield and Aquino, 1999; Aquino et al., 2001; Bordia et al., 2014). On the relationship between the hierarchical status and the staging of revenge, we were predicting that causal attributions (Peterson et al., 1982; Abramson et al., 1989; Marian and Filimon, 2010) the accusation leads with a low probability of revenge staging when the offending is in a position or has a higher status than the victim (without excluding equal or inferior status from the analysis). Direct revenge against a person of a higher status may cause retaliation because the offender of a high status may have a negative impact on the victim’s welfare than an offender of an equal or lower status (Sanders and Hamilton, 2001). Therefore, the fear of losing money, promotion opportunities, access to social support networks (Wilson et al., 2006) will influence the victim to defeat revenge (Post, 2008; Barattucci et al., 2017). The threat of loss of privileges is less important if the offending has an equal status (Aquino et al., 2001).

If the offending has a higher status than the victim, it is possible for the victim to decide that reconciliation is the best response. The victim will suppress their desire for revenge and abandon the negative emotions generated by the offense (Enright et al., 1989), as a result the employee will be more motivated to reconcile with the offending (McCullough et al., 1997, 1998). By restoring harmony in relationships, reconciliation pushes the victim’s own material interest forward by preserving opportunities for future rewards (House et al., 1988).

The set-up of revenge or reconciliation is preceded by a cognitive appreciation of the offense (Peterson et al., 1993), so in the case of retaliation, offense is perceived as dangerous (e.g., interpersonal abuse) or offense (e.g., breach of the rules). We believe that the appreciation of blame is the main driver of revenge which suggests that the offending is made responsible for the offense, in addition, the victim motivates a set of emotions such as anger and anger that increase the probability of revenge that activates the negative attributional patter.

As an extension of the blame-vengeance connection, the causal attributions are preachers of vengeance, but also of cognizance related to forgiveness or reconciliation. Most of the victim’s charges are attributed to the offender, when they frequently think of revenge and with a low frequency, the charges are attributed when the victim thinks about reconciliation.

## Conclusion

In this section, we will discuss the implications of our findings, strengths and limitations and propose further research directions.

Research has an interdisciplinary framework because it applies a hopelessness theory to understand an organizational phenomenon in a pandemic context. We therefore integrate attributional style, a widely used concept theoretically and applied with employee-related variables. This research contributes to strengthening a research path on labor and organizational health (House et al., 1988), highlighting the role of attributional style in training and maintaining employees’ attitudes and behavior.

As Ramkissoon (2021a) shows, a redeployment of work tasks and a re-establishment of the employee program is likely to reduce the impact on organizations and employees. On the other hand, we give credit to the claim that ”people suffering mental distress as a consequence of being laid off are likely to cultivate more negative place affect (p. 4).

Creativity will become a priority for companies and businesses around the world that are likely to focus on the potential of employees. Minh-Duc and Huu-Lam (2019) provided sound scientific data and further highlighted the inconsistency of current research examining the relationship between the intrinsic motivation and the creativity of employees.

Moreover, literature has largely examined the direct effect of attributional style on employee behavior (Henry, 2005; McCarter, 2007). Empirical studies are not enough to explain the mechanism by which employee behavior is influenced by attributional style and perception of social support. The results of the current study show that the way organizational communication works leads to trust or revenge among employees, who in turn influence their intentions and behavior. By explaining the basic mechanism of revenge and in part reconciliation, the overall implications of research are significant. Through our study we are supporting recent studies that address the influence of organizational communication in shaping employees’ attitudes at work.

As discussed earlier, the results have shown that different types of offenses have different influences the trust, orientation toward reconciliation or revenge of employees. This research suggests that attributional style and perception of social support have a different influence on employees’ behavior, as some employees focus on themselves, while others focus differently. These effects have previously been studied without continuity in this research direction. The fundamental idea of research will be useful for companies to formulate their communication strategies and mechanisms for eliminating conflicts.

We consider that social links have been affected in a negative sense, therefore, as Ramkissoon (2021b, p. 3) this appropriation is intended to cover the costs of the preparatory, technical and technical support, technical assistance and training measures necessary for the management of the operational program. Strengthening social links will become essential depending on the extent to which people will face separation and loss or other challenges encountered in the COVID-19 pandemic.

### Practical Implications

The information provided in this study has practical implications for organizations. Broadly speaking, we suggest the need to train employees’ top clerks to understand the dynamics, prevention and control of revenge.

Negative attributional style can be a symptom of uncertainty born from poor organizational communication or can be the result of the perception of broken promises.

More specifically, the perception of the offense is difficult to manage by the hierarchical superiors, yet managers can prevent violations of psychological contracts by intentionally setting, monitoring and fulfilling informal expectations. Actions in organizations should be considered as part of the continuous establishment of strong rules of procedural justice at work (Aquino et al., 2001).

The efforts of organizations should be directed toward addressing the levers that increase confidence.

On the other hand, effective collaboration between individuals, communities, businesses, non-profit organizations, public health directorates and governments could help minimize and mitigate the negative impact of the COVID-19 pandemic (Ramkissoon, 2020).

### Limitations and Future Research Directions

Our study sets out some limitations that need to be commented on, a first problem could be related to employee selection and experimental design. However, the literature shows that the road from blaming to revenge and reconciliation follows causal sequences which we support with the study implemented. Another problem identified could be reflected by the use of independent and dependant variables, but the circumstances analyzed reinforce the likelihood that the relationships observed were a function of the manufacturers concerned rather than a methodological trick. Therefore, in future research, we should take into account even the characteristics of the offensive episode, such as the social and professional context in which it occurs.

Hypothetical vengeance and reconciliation measurement are likely to be self-enhancement. In order to tackle these problems in future studies, data will need to be collected from far more sources. We have tried to prevent this limitation by using employees from different business sectors.

We have failed to provide sufficient evidence that the status variable has moderated the relationship between blame and revenge. The status variable is likely to be strongly moderated by the relationship between causal attributions and emotional intra-psychic processes. Another explanation could be that victims of offending do not choose revenge because the associated costs would be too high so they choose not to “do anything.”

We support the assumption that the victim of an offense when he cannot choose to take revenge will choose to behave in a reconcilable manner. Vengeance and reconciliation are not mutually and exhaustively adjustable responses (or coping) to perceived inequity. Therefore, we believe that they are rather mutual and exclusively copy responses.

We acknowledge that there may be other answers to perceived injustice apart from revenge and reconciliation. The aim of the study was not to draw up a conceptual map and measure all possible responses to perceived damage, but to research opposite actions such as reconciliation and revenge in the background crisis situation (the WHO-declared pandemic).

## Data Availability Statement

The raw data supporting the conclusions of this article will be made available by the authors, without undue reservation.

## Ethics Statement

The studies involving human participants were reviewed and approved by Ethics Committee for Research, Faculty of Socio-Humanistic Sciences, University of Oradea. The patients/participants provided their written informed consent to participate in this study.

## Author Contributions

MM: conceptualization, methodology, validation, and writing—original draft preparation. MM, KB, and MO: software, formal analysis, investigation, and writing—review and editing. KB and MO: visualization. All authors contributed to the article and approved the submitted version.

## Conflict of Interest

The authors declare that the research was conducted in the absence of any commercial or financial relationships that could be construed as a potential conflict of interest.

## Publisher’s Note

All claims expressed in this article are solely those of the authors and do not necessarily represent those of their affiliated organizations, or those of the publisher, the editors and the reviewers. Any product that may be evaluated in this article, or claim that may be made by its manufacturer, is not guaranteed or endorsed by the publisher.
